# Detection of postpartum women anemia and its impact on their offspring in Zhejiang Province, China

**DOI:** 10.3389/fnut.2025.1535226

**Published:** 2025-01-27

**Authors:** Mengjie He, Lichun Huang, Yan Zou, Peiwei Xu, Danting Su, Dong Zhao, Dan Han, Ronghua Zhang

**Affiliations:** Department of Nutrition and Food Safety, Zhejiang Provincial Center for Disease Control and Prevention, Hangzhou, China

**Keywords:** anemia, postpartum women, offspring, determinant, prenatal anemia

## Abstract

**Background:**

Information on the prevalence of postpartum women anemia is scarce in China, as well as its impact on their offspring. This study aimed to explore the influencing factors of anemia and the impact on the offspring.

**Methods:**

A cross-sectional study was conducted among 977 women within 2 years postpartum in 10 sites from 2016 to 2017 in Zhejiang Province, China. Information on demographics, nutrient supplementation and diet was collected. Whole blood hemoglobin, serum ferritin and transferrin receptor were assessed for all women and whole blood hemoglobin was assessed in offspring. The multivariate logistic regression was used to assess the determinants of anemia and the impact on the offspring by calculating odds ratio (OR) and its 95% confidence interval (95%CI).

**Results:**

Among 977 participants, 144 (14.74%) had anemia. Regression analysis showed that women intaking iron supplements during pregnancy (0.549, 0.350 ~ 0.860), and intaking sufficient red meat (0.647, 0.437 ~ 0.958) had lower rates of anemia, while women wo had anemia during pregnancy (2.754, 1.844 ~ 4.113 for mild anemia, and 3.815, 1.564 ~ 9.309 for moderate or severe anemia), had postpartum over 7 months (1.717, 1.112 ~ 2.650), with abnormal serum ferritin (11.931, 4.846 ~ 29.379) and excessive transferrin receptor levels (1.817, 1.050 ~ 3.145) had higher rates of anemia. Moreover, maternal anemia significantly increases the rate of anemia in offsprings, with ORs being 1.456 (0.994 ~ 2.133) for mild anemia, and 2.961 (1.242 ~ 7.058) for moderate or severe anemia, respectively.

**Conclusion:**

The status of anemia in postpartum women was severe and its impact on the anemia of their offspring should be noted. It is highly necessary to strengthen the regular monitoring of hemoglobin levels in pregnant and postpartum women, as well as implement targeted dietary interventions and suggest to intake dietary supplements if necessary.

## 1 Introduction

Anemia is widespread across in low- and middle-income countries (1, 2). As defined by the World Health Organization, anemia is characterized by a decrease in the proportion of red blood cells, a reduction in hemoglobin concentration levels, or an insufficient oxygen-carrying capacity to meet physiological requirements (3). A multiplicity of factors can precipitate anemia, with iron deficiency emerging as the predominant cause in the general populace. Globally, women consistently represent a high-risk cohort for this condition (1, 4, 5). According to an estimate by the World Health Organization (WHO), around, or even exceeding, half a billion women, equivalent to 29.9% of reproductive-age women (15–49 years) and 36.5% of pregnant women, were afflicted with anemia in 2019, with the vast majority suffering from iron deficiency (6). In China, the anemia problem among women is also acute (2). Reports suggest that the anemia prevalence among pregnant women in China from 2016 to 2020 was 41.27%, and the overall incidence of postpartum anemia reached 32.7% in 2019 (2, 7). Pregnant and postpartum women are especially susceptible to iron deficiency anemia due to the surging nutritional demands of the mother, fetus, and infant during these stages. Maternal health during pregnancy and lactation is directly correlated with the well-being of their offspring. Anemia exerts a deleterious impact on women of reproductive age and child health alike, which not only boosts morbidity and maternal mortality but also obstructs socioeconomic progress (5, 8, 9). Currently, numerous investigations (10–13) concentrate on anemia in pregnant women, given the high incidence of anemia among pregnant women and its direct impacts on the growth and development of the fetus or infant, such as low birth weight.

However, anemia in women within the two-year postpartum period, along with targeted, precise interventions, has remained grossly understudied. In reality, the problem of anemia among postpartum women remains grave (7). It is directly linked to postpartum recovery and can also affect the growth and development of infants by influencing the nutritional status of breast milk. An Indian study (14) found that 47.3% of women within 6 weeks postpartum were suffering from anemia. Therefore, greater attention should be directed toward postpartum women and their children, who are at elevated risk of anemia, to enable early identification, accurate diagnosis, and prompt treatment. Hence, the purpose of this study is to identify high-risk or early-warning groups for anemia by exploring the influencing factors of postpartum women’s anemia and the maternal-related influencing factors of offspring anemia. Ultimately, this will lay the foundation for formulating relevant intervention strategies to combat anemia in postpartum women and their offspring.

## 2 Materials and methods

### 2.1 Study design

This study was based on data obtained from the China National Nutrition and Health Survey 2016–2017 (CHNNS2016-2017) (15). The China National Nutrition and Health Survey conducted during 2016-mingx2017 was a cross-sectional study designed with the aim of investigating the health and nutritional status of children, adolescents, and women within 2 years postpartum. This study chose women within 2 years postpartum and their offspring from 10 investigation sites, accounting for one-tenth of the districts and counties, in Zhejiang Province, to form a representative provincial sample of Zhejiang Province to assess the nutritional status of postpartum women. A multi-stage stratified cluster sampling method was adopted for participant selection. In Zhejiang Province, there were 10 study sites that represented both urban and rural areas across the province. Subsequently, two townships or subdistricts were randomly selected from each study site. From the chosen townships or subdistricts, two villages or communities were randomly sampled. Finally, postpartum women within 2 years and their children under 2 years old, who resided in the selected villages or communities, were included and interviewed. At each survey site, at least 100 women and their corresponding children were interviewed. For this study, the inclusion criteria for the women and children were as follows: (1) agreed to participate in the study; (2) within 2 years after delivery (women) and their children less than 2 years old (children). The exclusion criteria were: (1) genetic metabolic disorders; (2) chronic cardiovascular and cerebrovascular diseases; (3) mental illnesses. The field surveys, physical examination and blood specimen collection were conducted in two phases, which were completed in September 2016 and November 2017, respectively. Each phase lasted for 2–3 months.

### 2.2 The ethical approval and consent form

The study was conducted in accordance with the Declaration of Helsinki and approved by the Institutional Review Board of the Chinese Center for Disease Control and Prevention (protocol code 201614 and approval date 3 June 2016). All women and children provided written informed consent after the research protocols were carefully explained to them.

### 2.3 Data collection

General information questionnaires were used to collect data regarding women’s age, nationality, educational level, occupation, parity, postpartum hemorrhage, the time when menstruation resumed, breastfeeding status, prenatal anemia, daily consumption of red meat and animal viscera, as well as iron supplementation during pregnancy and at present. Information about children’s age, their daily intake of red meat and animal viscera, and iron supplementation was also collected through these questionnaires. Physical examinations were carried out by trained health workers from the local community health center. Body Mass Index (BMI) was calculated by dividing weight (in kilograms) by the square of height (in meters), and then the subjects were categorized into three groups (16) (lean: <18.5; normal: 18.5–23.9; overweight: 24–27.9; obesity: ≥28 kg/m^2^).

### 2.4 Hemoglobin determination

Whole blood hemoglobin concentration was measured using Hemocue hemoglobin meter on the spot. Ten microliters of blood were collected from women’s fingertip and placed in the hemoglobin meter within 40 s. A quality control solution needs to be tested once whenever the machine was restarted or 20 to 30 samples was tested.

### 2.5 Blood sample collection and measurement

Six milliliters of fasting venous blood were drawn. Serum ferritin, transferrin receptor levels were measured by a fully automated analyzer based upon electrochemiluminescence and immunoturbidimetric assay method (Abbott, United States). Megaloblastic anemia, as a type of anemia, is related to folic acid and vitamin B12 deficiencies. Vitamin A malnutrition was reported to be related to iron deficiency anemia. Therefore, serum B12 and folate levels were measured by a fully automated analyzer based upon the chemiluminescence immunometric assay method (Abbott, United States). Vitamin A levels were measured by high performance liquid chromatography–tandem mass spectrometry (HPLC-MS/MS) method.

### 2.6 Definition of anemia and biomarkers

According to WHO standards (17), infants within 1 month with Hb < 145 g/L, infants aged 1–6 months with Hb < 90 g/L, children aged 6 months to <5 years with Hb < 110 g/L, children aged 5–11 years with Hb < 115 g/L, children aged 12–13 years and non-pregnant females over 14 years old with Hb < 120 g/L, were all defined as anemia. Anemia in adults was classified as Hb <80 g/L, Hb 80–100 g/L, and Hb 110–120 g/L as severe, moderate, and mild. Biomarkers included serum ferritin and serum transferrin receptor. Serum ferritin levels were divided into two groups: normal and inadequate (18), while transferrin receptor levels were classified into normal and excessive groups (19).

### 2.7 Statistical analyses

Mean and standard deviations (mean ± SD) were used to described continuous normal variables, and median and quartile (median (quartile)) were used for variables with skewed distribution. Frequency and percentage (%) were reported for the categorical variables. Continuous and categorical variables were compared using Student’s *t*-test and the chi-square test, respectively. A multiple logistic model was used to detect the association between postpartum time, BMI, iron supplement intake during pregnancy, red meat intake, serum vitamin A level, serum ferritin level, serum transferrin receptor level, anemia during pregnancy and incidence of anemia at present, respectively. These two models were also performed to investigate the relationship between influence factors in mothers and incidence of anemia in offsprings. Additionally, the figures were plotted based on Restricted Cubic Spline (RCS) Model. All statistical analyses were performed by using the program SAS version 9.1. *p* < 0.05 was considered statistically significant.

## 3 Results

### 3.1 Basic information

**Figure 1:**
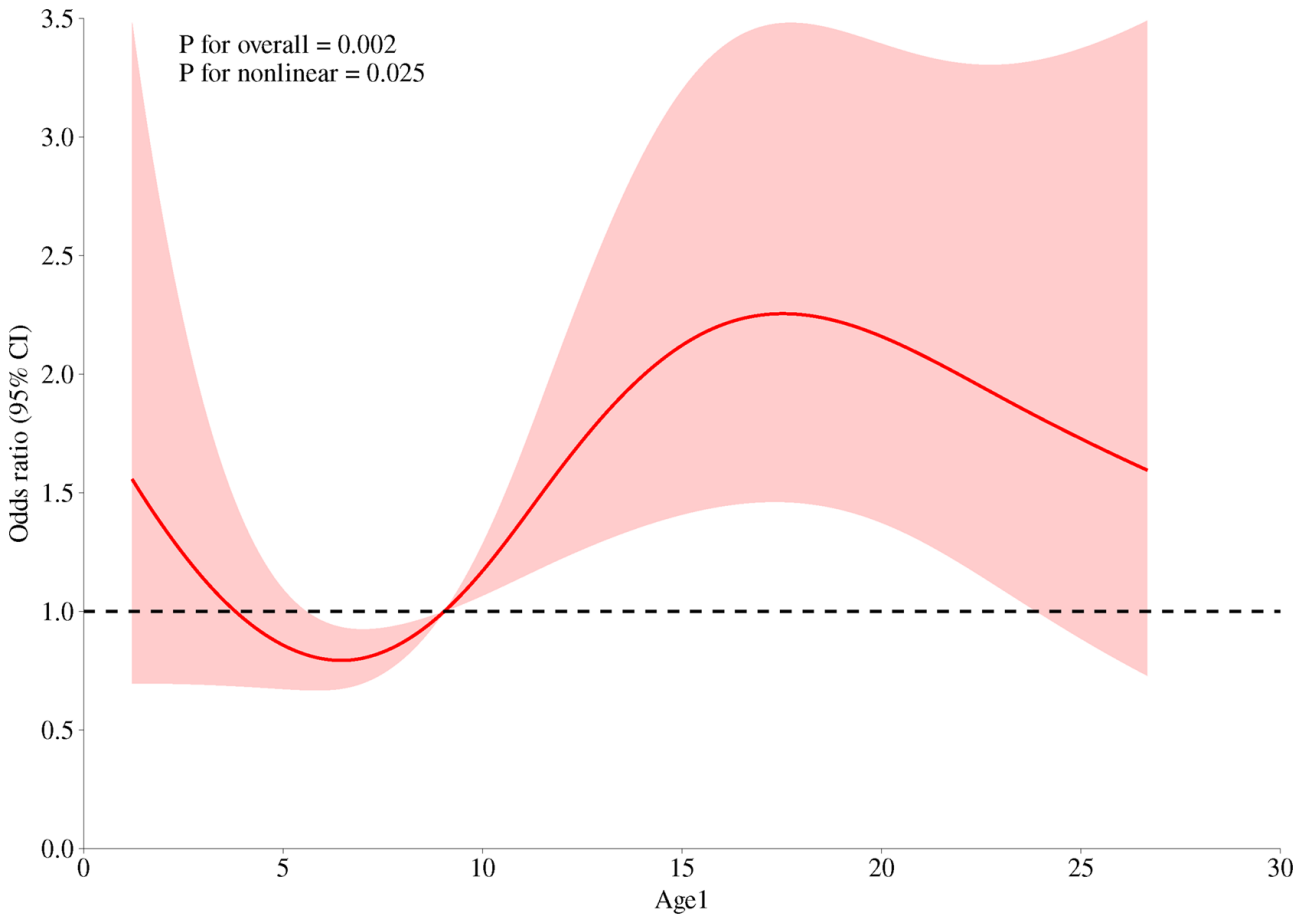
Relationship between continuous changes of postpartum durations and the occurrence of anemia among women within 2 years postpartum based on the RCS model.

A total of 977 women within 2 years postpartum were enrolled in the current study. The demographic characteristics of the participants are presented in Table 1. Overall, the prevalence of anemia of 977 postpartum women was 14.74%. The risk of anemia in postpartum women increased significantly after 9 months postpartum (see Figure 1). The basic information indicated that the included survey subjects were evenly distributed in age ranging from 20 to 40 years old, and the postpartum age was evenly distributed between 1 and 24 months. The nationality of the subjects was predominantly Han. The regional distribution, education level, and occupation were also evenly distributed. All of the above suggest that the survey subjects have good representativeness.

**Table 1:** Basic characteristics of women within 2 years postpartum.

Variables	Value
Participants (N)	977
Age of women, year^#^	
<30	468(47.9)
30 ~ 40	462(47.3)
40~	47(4.8)
Postpartum age, month^#^	
<7	370(37.9)
≥7	607(62.1)
Nationality^#^	
Han	947(98.1)
Others	18(1.9)
Educational level^#^	
Junior school or below	293(30.4)
Senior high school	443(45.9)
College or above	229(23.7)
Occupation^#^	
Intellectual work	416(43.1)
Physical work	483(50.1)
Retirement or unemployed	66(6.8)
BMI, kg/m^2#^	
<18.5	76(7.8)
18.5 ~ 24	608(62.5)
24.0 ~ 28.0	223(22.8)
28.0~	67(6.9)
Parity^#^	
1	470(48.3)
≥2	503(51.6)
Unknown	1(0.1)
Postpartum hemorrhage^#^	
Yes	25(2.6)
No	945(97.1)
Unknown	3(0.3)
Breastfeeding and weaning^#^	
Breastfeeding	427(43.9)
Breastfed and weaning	497(51.1)
Never breastfeed	48(4.9)
Iron supplement during pregnancy^#^	
No	649(66.6)
Yes	325(33.4)
Iron supplement during postpartum^#^	
No	943(96.5)
Yes	34(3.5)
Red meat intake^#^	
<50 g	424(43.4)
≥50 g	553(56.6)
Intake of animal viscera^#^	
Yes	744(76.7)
No	226(23.3)
Time of menstruation to resume, month^*^	5.63 ± 3.66
Serum ferritin μg/L^*^	67.56 ± 70.15
Serum transferrin receptor g/L^*^	2.54 ± 1.04
Vitamin A, pg/mL^*^	0.43 ± 0.13
Vitamin B12, pg/mL^*^	529.96 ± 224.68
Folic acid, ng/mL^*^	8.10 ± 3.96
Prenatal anemia^#^	
No	614(64.9)
Mild anemia	303(32.0)
Moderate to severe anemia	29(3.1)
Anemia^#^	
Yes	144(14.7)
No	833(85.3)

^*^Values are mean ± SD; ^#^ values are N (%).

The numbers with missing value were 2 for nationality, 12 for educational level, 12 for occupation, 3 for BMI, 3 for parity, 4 for postpartum hemorrhage, 5 for breastfeeding and weaning, 3 for iron supplement during pregnancy, 7 for intake of animal viscera, 31 for prenatal anemia, 59 for serum ferritin, 59 for serum transferrin, 59 for vitamin A, 59 for vitamin B12 and 59 for folic acid, respectively.

### 3.2 Status and determinants of iron deficiency anemia and iron reserve in postpartum women

It was shown in Table 2 that longer postpartum time (*p* = 0.012), overweight or obese (*p* = 0.013), no iron supplement intake during pregnancy (*p* = 0.008), less red meat intake (*p* = 0.014), lower serum vitamin A (*p* = 0.009), inadequate serum ferritin (*p* < 0.0001), excessive serum transferrin receptor (*p* < 0.0001) and anemia during pregnancy (*p* < 0.0001) were associated with increased risk of anemia in postpartum women. Multivariate analysis of statistically significant factors, postpartum time, BMI, iron supplement intake during pregnancy, red meat intake, serum vitamin A level, serum ferritin level, serum transferrin receptor level and anemia during pregnancy in univariate analysis showed that postpartum time, iron supplement intake during pregnancy, red meat intake, serum ferritin level, serum transferrin receptor level and anemia during pregnancy were associated with anemia in postpartum women (*p* < 0.05) in Table 3. The risk of anemia in postpartum women was higher with longer postpartum time (OR = 1.717, 95% CI = 1.112 ~ 2.650). Iron supplement intake during pregnancy (OR = 0.549, 95% CI = 0.350 ~ 0.860) and ≥ 50 g/d red meat intake (OR = 0.549, 95% CI = 0.350 ~ 0.860) were protective factors for anemia. Inadequate serum ferritin (OR = 11.931, 95% CI = 4.846 ~ 29.379), excessive serum transferrin receptor (OR = 1.817, 95% CI = 1.050 ~ 3.145) and anemia during pregnancy were risk factors for anemia.

**Table 2:** Univariate analysis of the risk of anemia in postpartum women.

Variables	Anemia, n (%)	Normal, n (%)	χ^2^	*P*
Total	144 (14.7)	833 (85.3)		
Region			0.005	0.944
	Urban	73 (51.1)	417 (50.7)	
	Rural	70 (49.0)	405 (49.3)	
Age of women, year			0.039	0.981
	<30	70 (48.6)	398 (47.8)		
	30 ~ 40	67 (46.5)	395 (47.4)		
	40~	7 (4.9)	40 (4.8)	
Postpartum age, month			6.341	0.012
	<7	41 (28.5)	329 (39.5)		
	≥7	103 (71.5)	504 (60.5)	
Educational level			1.467	0.480
	Junior school or below	44 (30.8)	249 (30.3)	
	Senior high school	60 (42.0)	383 (46.6)	
	College or above	39 (27.3)	190 (23.1)	
Job			0.079	0.962
	Intellectual work	62 (43.4)	354 (43.1)	
	Physical work	72 (50.4)	411 (50.0)	
	Retirement or unemployed	9 (6.3)	57 (6.9)	
BMI (kg/m^2^)			10.501	0.005
	≥18.5 ~ <24.0	99 (68.8)	509 (61.4)	
	<18.5	17 (11.8)	59 (7.1)	
	24~	28 (19.4)	261 (31.5)	
Parity			0.180	0.914
	1	70 (48.6)	400 (48.3)	
	≥2	74 (51.4)	429 (51.8)	
Postpartum hemorrhage			0.9728	0.615
	Yes	3 (2.1)	22 (2.7)	
	No	140 (97.2)	805 (97.1)	
	Unknown	1 (0.7)	2 (0.2)	
Breastfeeding and weaning			3.917	0.141
	Breastfeeding	73 (51.4)	492 (59.6)	
	Breastfed and weaning	59 (41.6)	296 (35.8)	
	Never breastfeed	10 (7.0)	38 (4.6)	
Iron supplement during pregnancy			8.3012	0.004
	No	111 (77.1)	538 (64.8)	
	Yes	33 (22.9)	292 (35.2)	
Iron supplement during postpartum			0.000	0.996
	No	139 (96.5)	804 (96.5)	
	Yes	5 (3.5)	29 (3.5)	
Red meat intake			6.049	0.014
	<50 g	76 (52.8)	348 (41.8)	
	≥50 g	68 (47.2)	485 (58.2)	
Intake of animal viscera			0.904	0.342
	Yes	38 (26.4)	188 (22.8)	
	No	106 (73.6)	638 (77.2)	
Serum ferritin μg/L			38.477	<0.0001
	Inadequate	27 (19.6)	38 (4.9)	
	Normal	111 (80.4)	742 (95.1)	
Serum transferrin receptor g/L			20.613	<0.0001
	Inadequate	31 (22.5)	72 (9.2)	
	Normal	107 (77.5)	708 (90.8)	
Prenatal anemia			24.812	<0.0001
	No	67 (47.2)	547 (68.0)	

**Table 3:** Analysis of the risk of anemia in postpartum women by multivariate logistic regression model.

Variables	β	s $\bar{\chi }$	Wald χ^2^	OR(95%CI)	*P*
Intercept	0.10	0.52	0.04		0.848
Postpartum age, month					
<7					
≥7	0.54	0.22	5.96	1.72 (1.11 ~ 2.65)	0.015
Iron supplement intake during pregnancy					
No					
Yes	−0.60	0.23	6.84	0.55 (0.35 ~ 0.86)	0.009
Red meat intake
<50 g					
≥50 g	−0.44	0.20	4.72	0.65 (0.44 ~ 0.96)	0.030
Serum ferritin, μg/L (18)					
Normal					
Inadequate	2.48	0.46	29.08	11.93 (4.85 ~ 29.38)	<0.001
Serum transferrin receptor, g/L (19)					
Normal					
Excessive	0.60	0.28	4.55	1.82 (1.05 ~ 3.15)	0.033
Prenatal anemia					
No					
Mild anemia	1.01	0.21	24.53	2.75 (1.84 ~ 4.11)	<0.001
Moderate or severe anemia	1.34	0.46	8.66	3.82 (1.56 ~ 9.31)	0.003

### 3.3 The influence on offspring anemia

Analysis of possible influencing factors of anemia in 968 infants aged 0–2 years was performed and showed in Table 4. Age of infant (*p* < 0.001), whether the mother intake iron supplement during pregnancy (*p* = 0.006), whether breastfeeding (*p* = 0.043) and whether the mother had anemia during pregnancy (*p* = 0.021) were associated with increased risk of anemia in infant. Multivariate analysis of above statistically significant factors, in Table 5, showed that the risk of anemia in infants will increase significantly after 6 months of age. Additionally, maternal anemia during pregnancy, especially moderate to severe anemia, was a risk factor for anemia in offspring. On the contrary, maternal intake of iron nutritional supplements during pregnancy was a protective factor for anemia in offspring. An interesting thing was found that breastfeeding may be an important risk factor for anemia in offspring.

**Table 4:** Univariate analysis of the risk of anemia in infant.

Variables	Anemia, n (%)	Normal, n (%)	χ^2^	*P*
Total	173(17.9)	795(82.1)		
Region			2.052	0.152
	Urban	97(56.1)	398(50.1)	
Age of infant, month	Rural	76(43.9)	397(49.9)	
			60.847	<0.001
Parity	<6	17(9.8)	272(34.2)	
	6 ~ 12~	103(59.5)	247(31.1)	
	12 ~ 24	53(30.6)	276(30.6)	
			1.644	0.200
	1	76(43.9)	391(49.3)	
	≥2	97(56.1)	402(50.7)	
Breastfeeding			6.283	0.043
	Breastfeeding	115(66.47)	450(56.60)	
	Breastfed and weaning	53(30.64)	302(37.99)	
	Never breastfeed	5(2.89)	43(5.41)	
Iron supplement during mother pregnancy			7.664	0.006
	No	131(75.7)	515(64.8)	
	Yes	42(24.3)	280(35.2)	
Iron supplement				
	No	171(98.8)	785(98.7)	0.012
	Yes	2(1.2)	10(1.3)	
Red meat intake			0.888	0.346
	No	68(45.0)	204(40.7)	
	Yes	83(55.0)	297(59.3)	
Intake of animal viscera			3.022	0.082
	No	104(68.9)	306(61.1)	
	Yes	47(31.1)	195(38.9)	
Maternal anemia			0.534	0.465
	Normal	138(83.1)	642(85.4)	
	Anemia	28(16.9)	110(14.6)	
Maternal anemia during pregnancy			7.747	0.021
	No	98(59.0)	501(66.6)	
	Mild anemia	58(34.9)	233(31.0)	
	Moderate to severe anemia	10(6.0)	18(2.4)	

**Table 5:** Analysis of the risk of anemia in offsprings by multivariate logistic regression model.

Variables		β	s $\bar{\chi }$	Wald χ^2^	OR(95%CI)	*P*
Intercept		−2.78	0.29	92.82		<0.001
Age of infant, month						
	<6					
	6 ~ 12~	2.04	0.30	47.25	7.67(4.29 ~ 13.71)	<0.001
	12 ~ 24	1.86	0.36	27.30	6.45(3.21 ~ 12.97)	<0.001
Breastfeeding						
	Breastfeeding					
	Breastfed and weaning	−0.79	0.25	10.26	0.46(0.28 ~ 0.74)	0.001
	Never breastfeed	−1.01	0.51	3.95	0.37(0.14 ~ 0.99)	0.047
Iron supplement during mother pregnancy						
	No					
	Yes	−0.51	0.20	6.14	0.60(0.40 ~ 0.90)	0.013
Maternal anemia during pregnancy						
	No					
	Mild anemia	0.38	0.19	3.72	1.46(0.99 ~ 2.13)	0.054
	Moderate or severe anemia	1.09	0.44	6.00	2.96(1.24 ~ 7.06)	0.014

## 4 Discussion

The issue of anemia during pregnancy has received extensive attention (9, 12, 20). However, the nutritional status and postpartum recovery of postpartum women are equally important and are related to the health of their offspring. As of now, few studies have focused on anemia in postpartum women and its influence on offspring. Moreover, the evidence from previous studies may be rather outdated, thus calling for an update (21–23). The results showed that taking iron supplements during pregnancy, sufficient intake of red meat, and normal serum ferritin and transferrin receptor levels were protective factors for anemia in postpartum women. Anemia during pregnancy and postpartum age > 7 months were risk factors for postpartum anemia. Maternal intake of iron supplements during pregnancy was a protective factor for anemia in offspring. Maternal anemia during pregnancy, age > 6 months, and breastfeeding were risk factors for offspring. This result was generally consistent with the research evidence (13, 14, 24) on the influencing factors of anemia in postpartum women available so far.

In the analysis of risk factors for anemia in postpartum women, existing study evidences (21, 22, 25–27) were referred to, and factors such as region, age, BMI, intake of iron nutritional supplements during pregnancy and postpartum, and anemia during pregnancy were included. The results indicated that taking iron supplements during pregnancy, sufficient intake of red meat, and normal serum ferritin and transferrin receptor levels were protective factors against anemia in postpartum women. Anemia during pregnancy was a major risk factor for postpartum anemia. As the RCS model demonstrated, the risk of anemia in postpartum women increased significantly after 9 months postpartum, which might be attributed to the resumption of menstruation after childbirth. Simultaneously, it is noteworthy that the risk of anemia in postpartum women significantly increased after 7 months postpartum, which might be closely related to the restoration of postpartum menstruation. This study suggested that the time for the resumption of postpartum menstruation was 5.63 ± 3.66 months, which was quite consistent with the time point when the risk of anemia increased. However, some results were not entirely consistent with previous study evidences (28, 29). There was no significant difference in the incidence of anemia among postpartum women in terms of urban–rural distribution, which may be due to the generally improved economic level. After the poverty alleviation campaign, anemia caused by malnutrition has been significantly improved, which has also been verified by similar studies (30). There was no statistical significance in the correlation between serum vitamin A level, serum vitamin B12 level, serum folic acid level, and anemia in postpartum women, indicating that the main types of anemia were iron deficiency anemia, and the incidence rates of vitamin B12-deficiency anemia and folate-deficiency anemia were relatively low (31). The number of postpartum women taking iron supplements or suffering postpartum hemorrhage was very small, which may be the reason why there was no statistical difference in the intake of iron supplements or postpartum hemorrhage in the results. In conclusion, this study found that taking iron supplements during pregnancy and appropriate intake of red meat can effectively prevent anemia. Additionally, postpartum women who were diagnosed with anemia during pregnancy or who are more than 9 months postpartum may be high-risk groups, and special attention should be paid to the occurrence of anemia. Combined with the evidence from previous studies (32), serum ferritin and transferrin receptor, which reflect iron reserves, can provide early warning of the occurrence of anemia.

In the analysis of the factors influencing anemia in offspring, existing research evidences (33) were referred to, and factors such as region, age, parity of the mother, whether breastfeeding, iron nutritional supplement intake of infants and their mothers during pregnancy, intake of red meat and animal viscera, current or past maternal anemia during pregnancy, and serum ferritin and transferrin receptor levels of the mother were included. The results demonstrated that maternal intake of iron supplements during pregnancy was a protective factor for anemia in offspring. Maternal anemia during pregnancy, age > 6 months, and breastfeeding were risk factors for offspring. It was found that the risk of infant anemia significantly increased after 6 months after birth, which may be related to the fact that iron reserves at birth are only sufficient to cover the iron demands for growth during the first 4–6 months of life (34). Through an analysis of how the influencing factors of mothers during the postpartum stage and pregnancy affect the anemia of their offspring, it was discovered that the former has a more significant role. This might be attributed to the fact that the accretion of iron in the fetus mainly occurs during the third trimester of pregnancy (33). The results showed that iron supplement intake during pregnancy and diagnosed anemia during pregnancy had a more significant impact on the risk of anemia in offspring, which suggests that more attention should be paid to the iron nutritional status and anemia prevention of pregnant women during the fetal period. An Ethiopian study (35) showed that the government had already attached great importance to iron supplementation for pregnant women and was committed to improving adherence to iron supplementation. Additionally, an interesting finding was that breastfeeding actually increased the risk of anemia in offspring. Human milk is the ideal food for infants due to its unique nutritional characteristics. However, iron in it decreases progressively from day 1 to 14 weeks and at 6 months in both nonanemic and anemic mothers, and breastmilk iron and lactoferrin concentration have no relationship with the mother’s Hb and iron status (36). Therefore, it reminds us to pay attention to the intake of iron supplements for breastfed infants.

Our study has several strengths. First, it evaluated the status of anemia in both women during pregnancy and within 2 years postpartum as well as in their offspring. In contrast, other studies have only focused on anemia in pregnant or lactating women or children. At the same time, this study simultaneously compared the influences of maternal factors during pregnancy and postpartum on anemia in offspring. Second, the present study demonstrated that iron supplementation during pregnancy is an important factor for preventing anemia in postpartum women and their offspring. Third, 9 months postpartum and 6 months after birth may be high-risk time points for the occurrence of anemia in postpartum women and infants. Finally, it was found that breastfeeding might be a risk factor for infant anemia, suggesting that attention should be paid to early iron supplementation for breastfed infants.

However, this study has the following limitations, and caution should be exercised when interpreting and extrapolating the results. First, a cross-sectional design was adopted in this study. As a result, this design can only suggest possible influencing factors for maternal anemia and cannot clarify causal relationships. Second, information such as the diagnosis of anemia during pregnancy, nutrient supplementation, and dietary data were self-reported and might have been influenced by recall bias. Third, the anemia status of pregnant women may vary at different periods and under different interventions. Since this study only selected a single sample, it cannot objectively present the dynamic change process. Future studies can collect the results of multiple blood samples as well as information on interventions. Finally, the overall anemia rate of the patients included in the study was relatively low, and the sample size was limited, resulting in relatively wide confidence intervals. Therefore, it is still necessary to conduct larger-scale studies in the future.

## 5 Conclusion

To summarize, the status of anemia in postpartum women was severe and worthy of attention, meanwhile, its impact on the anemia of their offspring also alerted us. During pregnancy, ensuring sufficient consumption of red meat along with regular intake of iron supplements is crucial. Actively preventing and treating anemia throughout pregnancy is also of great significance. Meanwhile, close attention should be paid to biomarkers, including ferritin and transferrin receptor, which serve as reliable indicators of iron reserves. Such comprehensive measures can effectively mitigate the risk of postpartum anemia. The risk of anemia in postpartum women may increase after 7 months postpartum. In offspring, the fetal stage exerts a more profound impact on the risk of infantile anemia than the postnatal period. Concurrently, special attention ought to be directed toward iron supplementation in infants aged over 6 months or those who are breastfed. This targeted approach facilitates the timely prevention and treatment of anemia.

## Data Availability

The raw data supporting the conclusions of this article will be made available by the authors, without undue reservation.
